# Dental Status and 30-Day Postoperative Pneumonia in Burn Surgery Patients

**DOI:** 10.3390/medicina62081506

**Published:** 2026-08-05

**Authors:** Jihion Yu, Hee Yeong Kim, Young Joo Seo, Young-Kug Kim

**Affiliations:** 1 Department of Anesthesiology and Pain Medicine, Asan Medical Center, University of Ulsan College of Medicine, Seoul 05505, Republic of Korea; jihionyu@amc.seoul.kr (J.Y.); umelly98@gmail.com (H.Y.K.); 2 Department of Anesthesiology and Pain Medicine, Hallym University Hangang Sacred Heart Hospital, Seoul 07247, Republic of Korea

**Keywords:** burn surgery, preoperative dental status, postoperative pneumonia

## Abstract

*Background and Objectives*: Major burn injury causes systemic immune dysregulation and increases susceptibility to infectious complications. While poor oral health is a potential reservoir for respiratory pathogens, its impact on burn patients—who often require repeated airway manipulation—is not well-defined. This study investigated the association between preoperative dental status and postoperative pneumonia in patients undergoing burn surgery. *Materials and Methods*: We conducted a retrospective cohort study of 894 adult patients (≥19 years) admitted to a burn intensive care unit (ICU) between 2015 and 2024. Preoperative dental status was categorized as favorable or unfavorable (≥2 missing teeth, loose teeth, or fractured teeth). The primary outcome was 30-day postoperative pneumonia. Secondary outcomes included 90-day hospital-free days and ICU-free days. *Results*: Postoperative pneumonia occurred in 294 patients (32.9%). The incidence of postoperative pneumonia was 47.7% among patients with unfavorable preoperative dental status, compared with 31.2% among those with favorable preoperative dental status (*p* < 0.001). In multivariable analysis, unfavorable dental status was an independent predictor of 30-day pneumonia (odds ratio, 1.681; 95% confidence interval, 1.084–2.599; *p* = 0.018). Patients with unfavorable status also had significantly fewer 90-day hospital-free days (9 vs. 22 days, *p* = 0.034) and ICU-free days (65 vs. 68 days, *p* = 0.012). *Conclusions*: Unfavorable preoperative dental status is independently associated with an increased risk of postoperative pneumonia and poorer clinical recovery in burn patients. Routine dental assessment may serve as a simple tool for perioperative risk stratification in patients undergoing burn surgery.

## 1. Introduction

Major burn injury induces a profound hypermetabolic response and systemic immune dysregulation, which significantly increases the patient’s susceptibility to various infectious complications [1,2]. Among these, postoperative pneumonia remains one of the most prevalent and clinically burdensome complications, often leading to prolonged mechanical ventilation, extended intensive care unit (ICU) stays, and increased mortality rates in burn populations [3]. Despite advancements in critical care and surgical techniques, identifying modifiable risk factors for respiratory infections in major burns continues to be a major clinical challenge [4].

The oral cavity serves as a significant reservoir for opportunistic respiratory pathogens, and poor oral health—characterized by dental caries, periodontal disease, or heavy plaque accumulation—has been recognized as a potential source of aspiration pneumonia in critically ill patients [5]. In the context of major burns, patients often require endotracheal intubation and prolonged immobilization [3], which may further facilitate the translocation of oral bacteria into the lower respiratory tract. Furthermore, the systemic inflammatory milieu following a major burn may exacerbate existing oral infections, creating a bidirectional link that compromises overall clinical outcomes [1].

Although the association between poor oral health and pneumonia has been well established in geriatric [5,6,7] and critically ill populations [8,9], it has not been adequately investigated in patients with major burns. Several validated oral health assessment tools, including the Decayed, Missing, and Filled Teeth (DMFT) index, the Community Periodontal Index (CPI), and the Oral Health Assessment Tool (OHAT), have been developed for structured oral health evaluation [10,11,12]. However, the routine use of these instruments may be challenging in critically ill burn patients undergoing urgent surgery because comprehensive dental examinations are often impractical in this setting. Therefore, a simple and clinically applicable preoperative dental assessment may provide a feasible approach for perioperative risk stratification. Evaluating the association between preoperative dental status and postoperative pneumonia may help identify patients at increased risk for postoperative pulmonary complications and improve risk stratification in patients with major burns.

Therefore, this study investigated whether preoperative dental status was associated with postoperative pneumonia in adult patients undergoing surgery for major burns. We hypothesized that poor preoperative dental status would be independently associated with an increased risk of postoperative pneumonia and adverse postoperative outcomes.

## 2. Materials and Methods

### 2.1. Study Design and Patients

We conducted a retrospective cohort study at Hangang Sacred Heart Hospital, a tertiary care university hospital affiliated with Hallym University. Adult patients (≥19 years) admitted to the burn ICU who underwent burn surgery between 2015 and 2024 were included. Eligibility for burn ICU admission followed institutional criteria: deep burn area ≥ 20%; deep burn area ≥ 10% in elderly patients (≥65 years); high-voltage electrical burns; burn injuries accompanied by significant trauma or medical comorbidities; or inhalation injury [13]. Patients were excluded if medical records were incomplete or if they were transferred to another facility after the initial surgery.

### 2.2. Ethical Consideration

This study was approved by the Institutional Review Board of Hangang Sacred Heart Hospital (IRB No. 2026-010). The requirement for informed consent was waived due to the retrospective and observational nature of the study.

### 2.3. Data Collection

**Figure 1:**
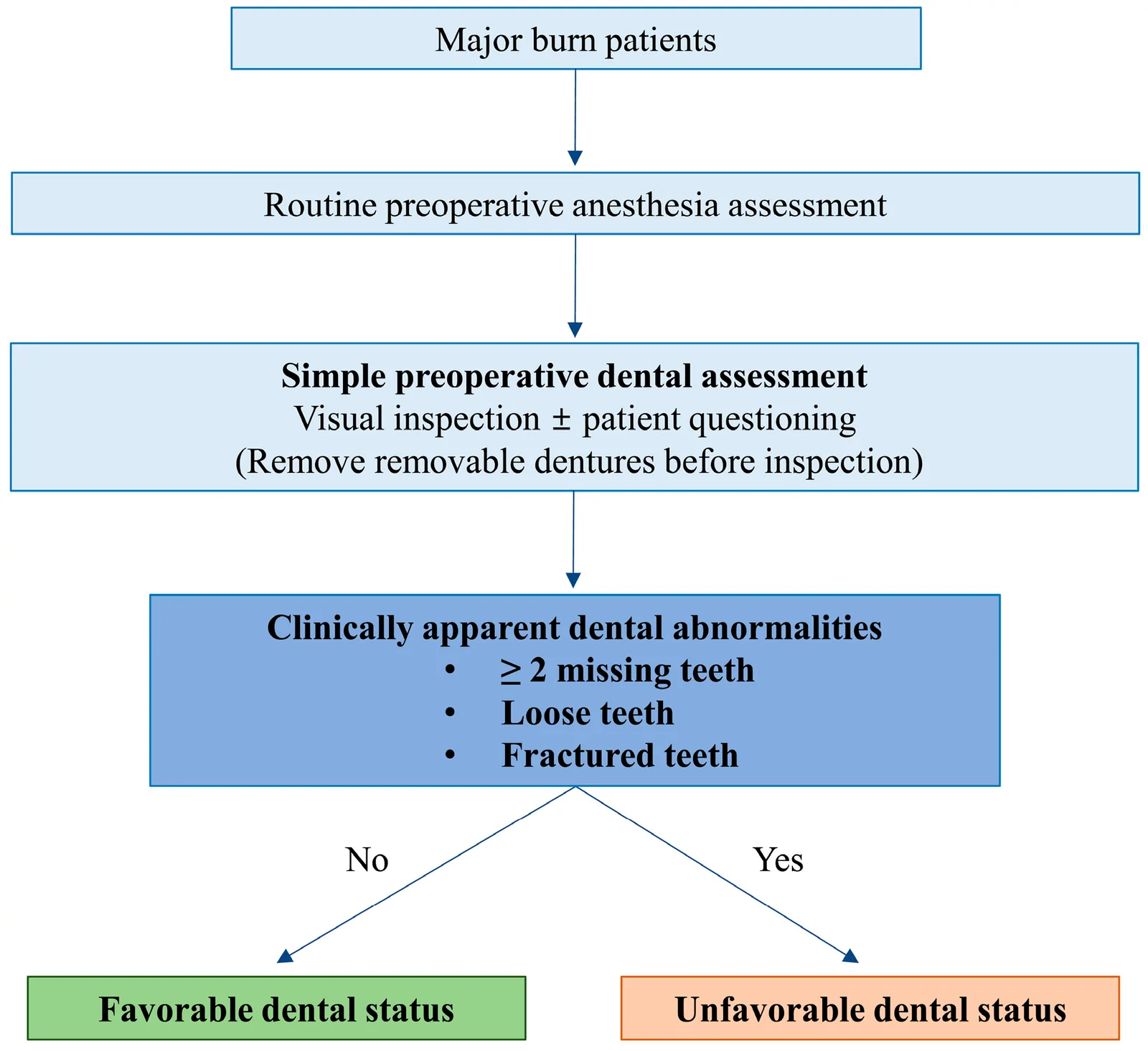
Practical framework for simplified preoperative dental assessment in patients undergoing major burn surgery. Unfavorable dental status was defined as the presence of two or more missing teeth, loose teeth, or fractured teeth identified during the routine preoperative anesthesia evaluation. The assessment was limited to these predefined findings and did not include a comprehensive dental or periodontal examination.

Clinical data were retrieved from the institutional clinical data warehouse, which integrates electronic medical records, pharmacy data, and laboratory databases. Collected variables included demographic characteristics; medical comorbidities (diabetes mellitus, hypertension, and end-stage renal disease); burn-related variables (burn mechanism, presence of inhalation injury, deep burn area, and post-burn day); preoperative dental status; laboratory data; and operative variables, including operative duration, vasopressor use, red blood cell (RBC) transfusion, volumes of crystalloid and colloid administration, and urine output. Inhalation injury was diagnosed based on bronchoscopy findings, clinical features, burn mechanism, and carboxyhemoglobin levels [14]. Deep burn area was estimated by experienced burn surgeons using a ruler-based method, based on the extent of deep second-degree or deeper burns [14]. Preoperative laboratory values, including hemoglobin, albumin, and creatinine, were collected within one day before surgery. Preoperative dental status was assessed and documented by experienced attending anesthesiologists as part of the routine preoperative anesthesia evaluation before induction of general anesthesia (Figure 1). These anesthesiologists routinely performed preoperative airway and oral cavity assessments as part of standard anesthesia practice. Dental findings were documented using the institution’s standardized preoperative anesthesia evaluation form, which was routinely used for all surgical patients. The assessment consisted of a brief visual inspection of the oral cavity, supplemented by patient questioning regarding dental status when feasible. In patients with impaired consciousness or inability to communicate, dental status was assessed solely by visual inspection. Removable dentures were removed before oral inspection, and classification was based on the underlying dentition. Dental status was categorized as favorable or unfavorable according to predefined study criteria established before data collection. Unfavorable dental status was defined as the presence of two or more missing teeth (including complete edentulism), loose teeth, or fractured teeth. Comprehensive oral health indicators, including periodontal disease, gingivitis, dental plaque, dental caries, oral hygiene, and gingival bleeding, were not systematically assessed.

### 2.4. Endpoints

The primary outcome was 30-day postoperative pneumonia. Secondary outcomes were hospital-free days and ICU-free days within 90 days. Pneumonia was defined based on burn-specific clinical diagnostic criteria [15]. The diagnosis required at least two of the following findings: (1) a new or persistent infiltrate, consolidation, or cavitation on chest radiography; (2) sepsis; or (3) a recent change in sputum production or purulence. Microbiologic data, when available, were used as supportive information but were not required for the diagnosis. Diagnoses that could mimic pneumonia, such as acute respiratory distress syndrome, tracheobronchitis, or chest contusion, were clinically excluded. Hospital-free days and ICU-free days within 90 days were defined as the number of days alive and not hospitalized or admitted to the ICU, respectively, during the first 90 postoperative days. Patients who died within 90 days were assigned zero free days [16].

### 2.5. Burn Management in ICU

All patients admitted to the burn ICU received standardized multidisciplinary care provided by burn surgeons, intensivists, and specialized therapists. Initial fluid resuscitation was performed according to the Parkland formula (4 mL/kg per percent total body surface area burned). Additional fluids were administered to maintain a target urine output of at least 0.5 mL/kg/h. Enteral nutrition was initiated within 48 h of admission in the absence of contraindications. Wound care consisted of routine cleansing followed by topical antimicrobial therapy and hydrofoam dressings, and was performed daily. Airway management was performed according to clinical indications [17]. Endotracheal intubation was performed in patients with airway compromise, severe inhalation injury, or decreased consciousness. In patients with preserved consciousness and without significant airway edema, intubation was avoided whenever possible. Mechanical ventilation was applied according to institutional ICU protocols when respiratory support was required. Routine surveillance for infection was performed through clinical assessment, laboratory testing, and microbiological cultures when indicated [15].

### 2.6. Intraoperative Practice

General anesthesia was administered according to standardized institutional protocols. Crystalloid solutions were infused at rates of 6–10 mL/kg/h. Synthetic colloids were administered when estimated blood loss exceeded 500 mL. RBC transfusion was initiated when hemoglobin levels decreased below 8 g/dL. Additional fluids or vasoactive agents were administered to maintain a mean arterial pressure ≥ 65 mmHg.

Surgical management prioritized early excision and grafting using autografts and/or allografts. Escharotomy or fasciotomy was performed when indicated to relieve elevated interstitial pressure. In cases of suspected deep tissue infection, extensive wound debridement was followed by application of silver sulfadiazine–impregnated dressings or negative-pressure wound therapy [18].

### 2.7. Statistical Analyses

Continuous variables are presented as medians with interquartile ranges. Between-group comparisons were performed using the independent t-test or the Wilcoxon rank-sum test, depending on data distribution. Categorical variables are expressed as frequencies and percentages and were compared using the chi-square or Fisher’s exact test. Multivariable logistic regression analysis was performed to evaluate the association between preoperative dental status and 30-day postoperative pneumonia. Variables were selected for the multivariable logistic model based on clinical relevance and univariate differences between the pneumonia and non-pneumonia groups. Variables demonstrating multicollinearity (variance inflation factor > 10) were excluded. Kaplan–Meier curves were constructed to compare the cumulative incidence of pneumonia within 30 days after surgery according to preoperative dental status, and differences between groups were assessed using the log-rank test. Continuous outcomes with non-normal distributions, including hospital-free days and ICU-free days within 90 days, were compared using the Mann–Whitney U test. All statistical analyses were performed using R software (version 4.3.2; R Foundation for Statistical Computing, Vienna, Austria) and SPSS Statistics for Windows (version 27.0; IBM Corp., Armonk, NY, USA).

## 3. Results

**Figure 2:**
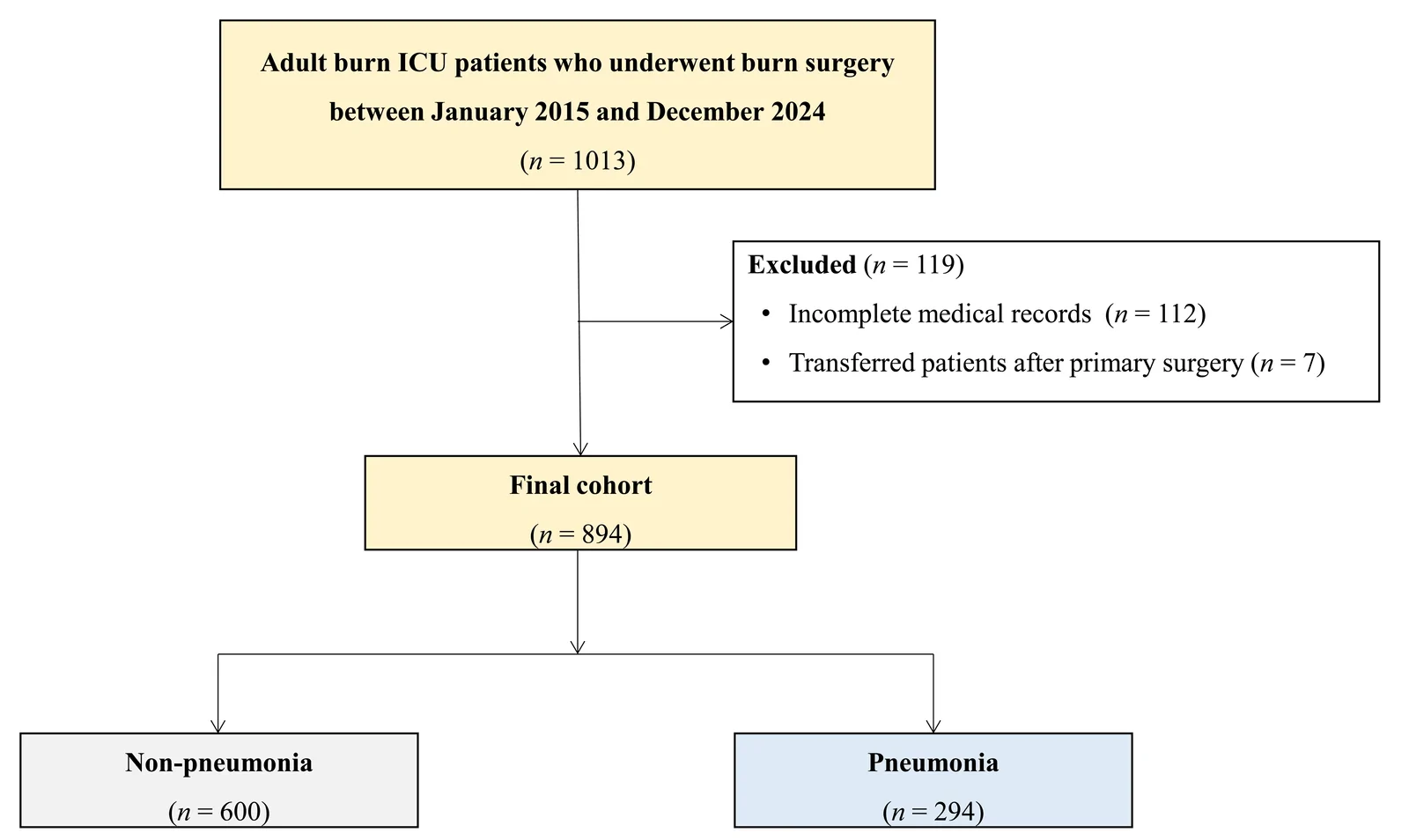
Flowchart of the study patients. Patients were dichotomized into non-pneumonia and pneumonia group. ICU, intensive care unit.

Among 1013 adult patients admitted to the burn ICU for burn surgery between January 2015 and December 2024, 119 were excluded because of incomplete medical records (*n* = 112) or transfer to another hospital after the initial surgery (*n* = 7). The final study cohort therefore consisted of 894 patients (Figure 2).

Postoperative pneumonia within 30 days occurred in 294 of 894 patients (32.9%). Baseline characteristics, burn severity, preoperative dental status, preoperative laboratory findings, and intraoperative variables were compared between patients with and without postoperative pneumonia (Table 1). Patients who developed postoperative pneumonia were older than those without pneumonia (58 vs. 52 years) and had a higher prevalence of inhalation injury (41.8% vs. 24.0%). In addition, they had a greater extent of deep burn area (35.0% vs. 28.0%), indicating more severe burn injury.

**Figure 3:**
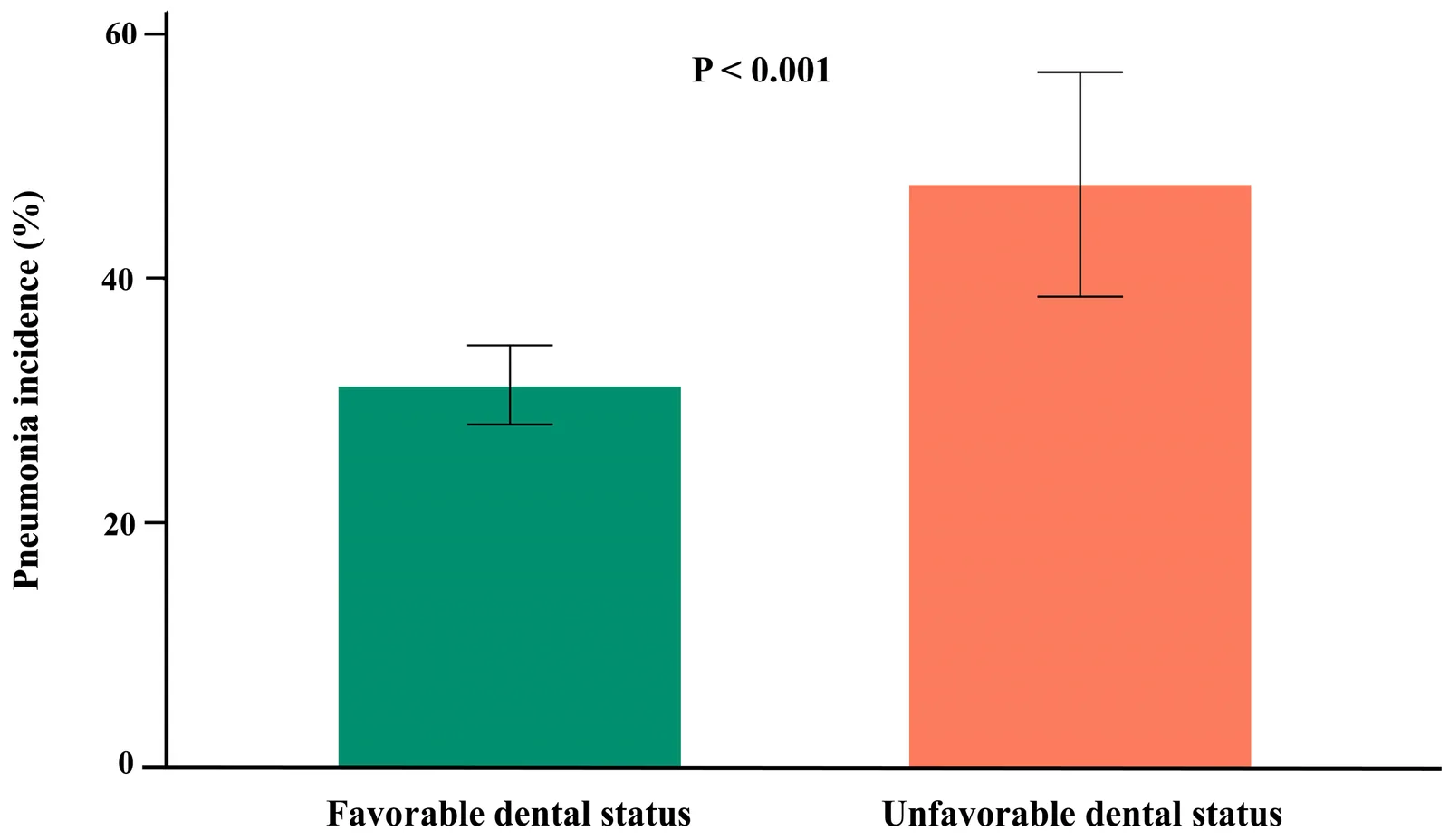
Incidence of postoperative pneumonia according to preoperative dental status. Patients with unfavorable dental status had a higher incidence of postoperative pneumonia than those with favorable dental status (47.7% vs. 31.2%, *p* < 0.001). Error bars indicate standard errors.

In multivariable logistic regression analysis, unfavorable dental status remained independently associated with 30-day postoperative pneumonia after adjustment for clinically relevant covariates, including age, sex, American Society of Anesthesiologists physical status, inhalation injury, deep burn area, and preoperative serum albumin and creatinine (Table 2). The incidence of postoperative pneumonia was significantly higher among patients with unfavorable preoperative dental status than among those with favorable preoperative dental status (47.7% vs. 31.2%, *p* < 0.001) (Figure 3).

**Figure 4:**
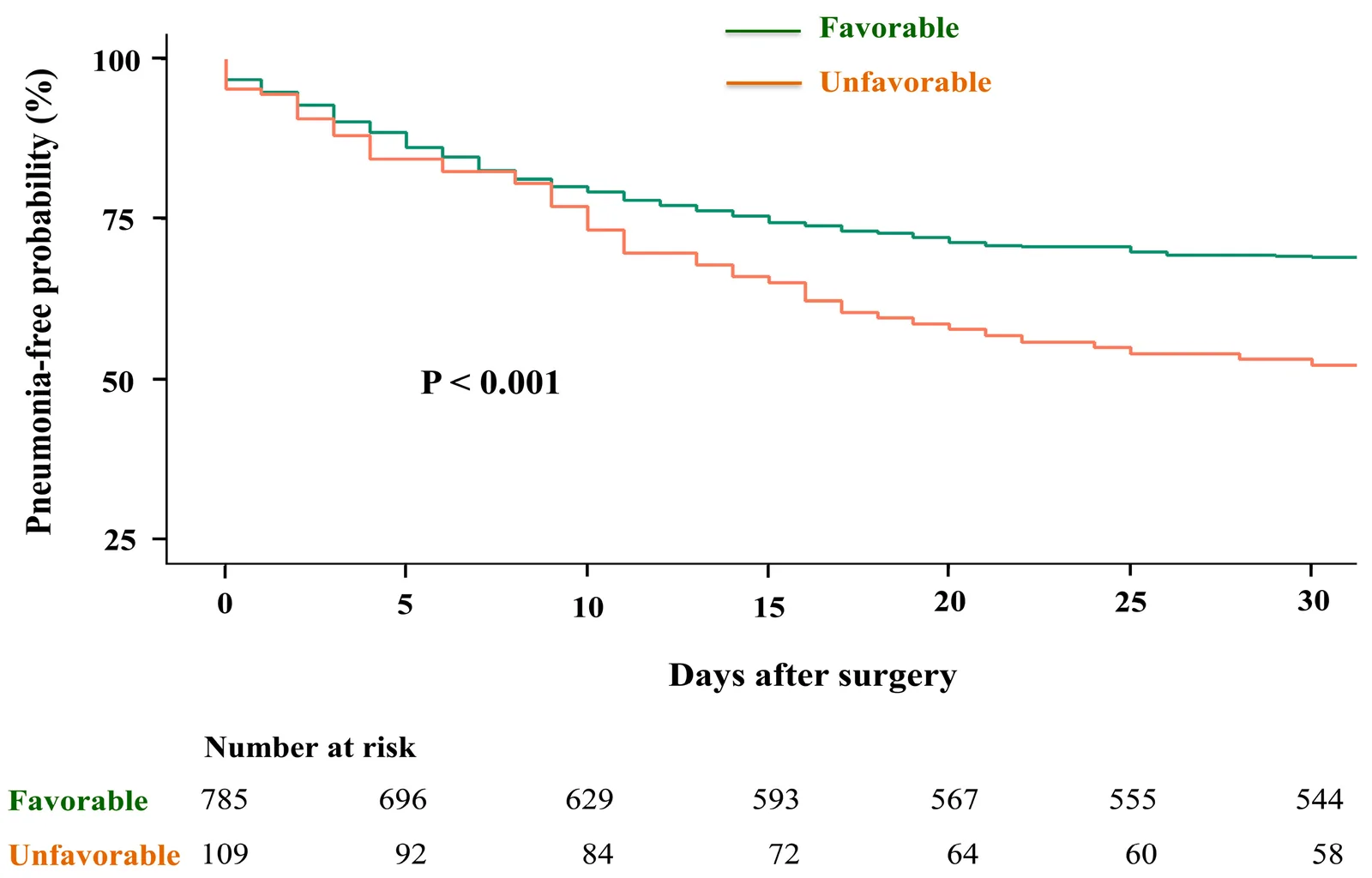
Kaplan–Meier curves showing the probability of remaining pneumonia-free within 30 days after burn surgery according to preoperative dental status. Patients with unfavorable dental status had a significantly lower pneumonia-free probability than those with favorable dental status (log-rank *p* < 0.001).

Kaplan–Meier curves demonstrated an early and sustained separation between patients with favorable and unfavorable dental status, with the latter showing a significantly lower pneumonia-free probability throughout the 30-day postoperative period (log-rank *p* < 0.001) (Figure 4).

**Figure 5:**
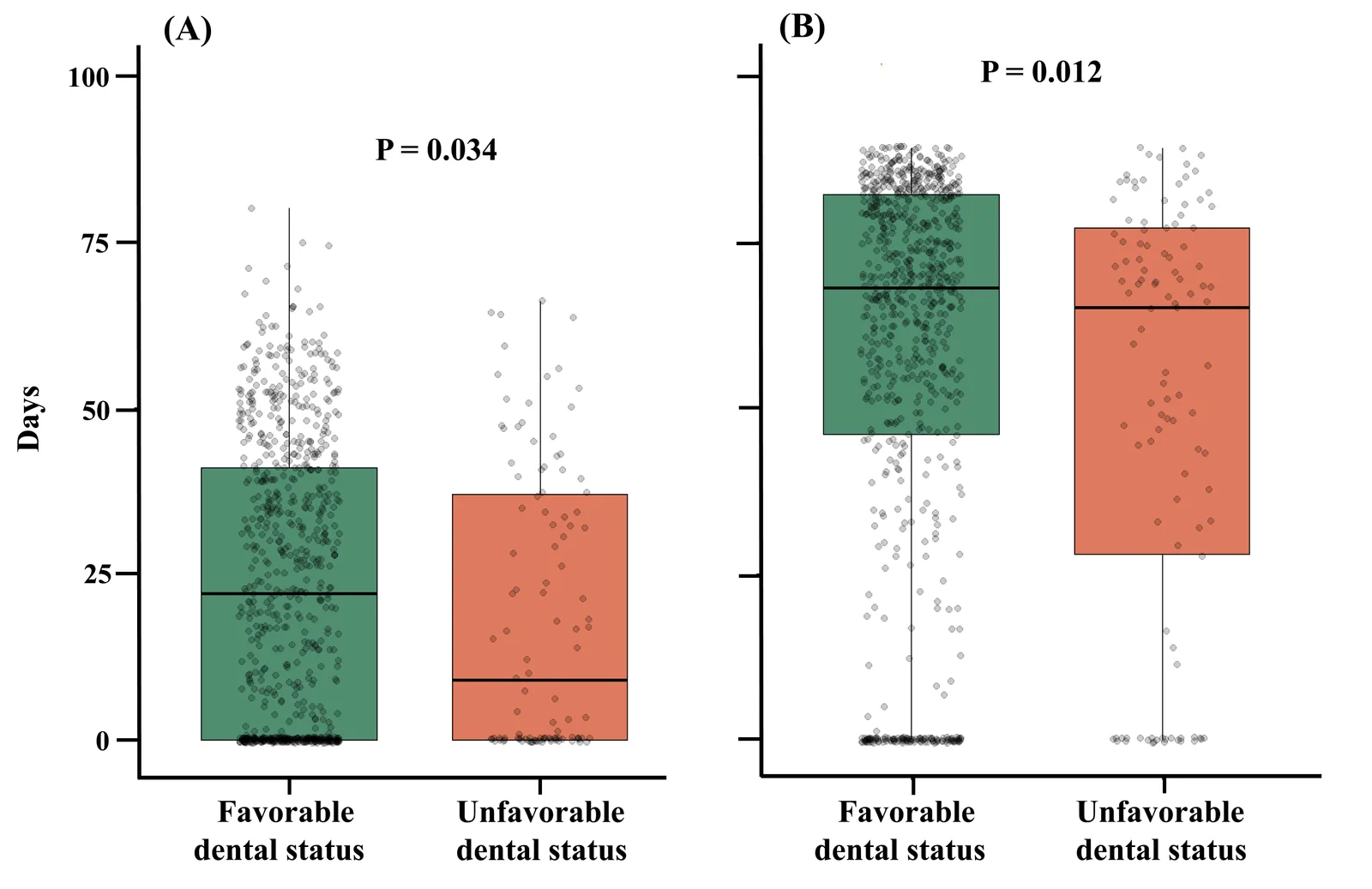
Comparison of hospital-free and ICU-free days within 90 days according to preoperative dental status. (**A**) Hospital-free days were lower in patients with unfavorable dental status than in those with favorable status (*p* = 0.034). (**B**) ICU-free days were lower in patients with unfavorable dental status than in those with favorable status (*p* = 0.012). Data are presented as box-and-whisker plots, with the center line indicating the median, the box representing the interquartile range, the whiskers showing the spread of the data according to the standard boxplot convention, and each dot representing an individual patient. ICU, intensive care unit.

Patients with favorable dental status had significantly more hospital-free days within 90 days than those with unfavorable dental status (22 [0–41] days vs. 9 [0–37] days, *p* = 0.034; Figure 5A). Similarly, ICU-free days within 90 days were higher in patients with favorable dental status compared with those with unfavorable dental status (68 [46–82] days vs. 65 [28–77] days, *p* = 0.012; Figure 5B).

## 4. Discussion

The present study demonstrated that preoperative dental status was independently associated with the risk of postoperative pneumonia in patients undergoing burn surgery. Patients with unfavorable dental status had a significantly higher incidence of pneumonia within 30 days after surgery and showed a lower pneumonia-free probability on Kaplan–Meier analysis. In addition, unfavorable dental status was associated with fewer hospital-free days and ICU-free days within 90 days. These findings suggest that dental status may represent an overlooked clinical factor associated with postoperative infectious complications in burn patients.

Patients with major burns have a markedly increased susceptibility to infection due to multiple pathophysiological factors [1,3]. Extensive burn injury disrupts the skin barrier, induces systemic inflammatory responses, and leads to immune dysregulation [1]. In addition, many burn patients require repeated surgical procedures and prolonged intensive care, which further increases exposure to invasive devices and nosocomial pathogens [19,20]. Respiratory complications, particularly pneumonia, are among the most frequent and clinically significant infections in this population and are associated with prolonged hospitalization [3,20,21]. These characteristics make the identification of modifiable risk factors particularly important in burn care.

Oral health has been increasingly recognized as a key contributor to systemic infections in the broader critically ill population [6]. Previous studies have established that inadequate oral hygiene increases the bacterial load in the oropharynx, thereby elevating the risk of ventilator-associated pneumonia in general ICU patients [9,22]. However, in the specific context of major burns, this risk may be further amplified by repeated airway manipulation. As burn patients frequently undergo multiple surgical procedures requiring repeated endotracheal intubation [19], oral microorganisms are more readily introduced into the lower respiratory tract [23]. Despite these clinical concerns, the prognostic relevance of dental status in burn patients remains poorly defined, highlighting the need for a simple and clinically applicable perioperative assessment.

Several validated instruments, such as OHAT, provide comprehensive evaluations of multiple oral domains, including the teeth, oral mucosa, saliva, oral cleanliness, dentures, and oral pain. However, such multidomain assessments may be difficult to implement retrospectively or in critically ill patients with impaired consciousness or limited cooperation [11]. Similarly, although the CPI is a standardized and validated periodontal assessment tool, it requires a dedicated periodontal examination that was not routinely documented in retrospective perioperative anesthesia records and was therefore not feasible for use in the present study [24]. Accordingly, the dental classification used in this study was intended as a pragmatic perioperative screening approach rather than a substitute for a validated oral health index. We acknowledge that the presence of two missing teeth does not necessarily reflect poor overall oral health; rather, this threshold was selected as a pragmatic screening criterion for retrospective perioperative assessment rather than as a comprehensive measure of oral health. It was based on visual inspection and brief patient questioning and required no specialized dental equipment. The threshold of two or more missing teeth was prespecified before study initiation to improve the consistency and reproducibility of retrospective classification because isolated tooth loss was inconsistently documented in routine anesthesia records, whereas multiple missing teeth and complete edentulism were more reliably identifiable. Future prospective studies should directly compare this simplified assessment with validated oral health instruments.

Several mechanisms may explain the association between unfavorable dental status and postoperative pneumonia. The oral microbiome is increasingly recognized as an important reservoir of opportunistic respiratory pathogens, and oral microbial dysbiosis has been associated with the development of pneumonia and poor clinical outcomes in critically ill patients [25,26]. Oral dysbiosis may increase colonization by potentially pathogenic microorganisms, thereby facilitating aspiration-related pulmonary infection during repeated airway manipulation and perioperative care. Accordingly, damaged or missing teeth may further promote bacterial retention within the oral cavity [9,23]. During anesthesia or postoperative recovery, microaspiration of oral secretions may allow these organisms to enter the lower respiratory tract [7]. In addition, poor oral health may reflect underlying frailty or reduced self-care ability [27,28,29], which could impair airway protection and clearance of respiratory secretions. In burn patients who frequently undergo repeated anesthesia and prolonged hospitalization [2], these mechanisms may further increase susceptibility to pulmonary infection [20]. Beyond its conventional role in preventing dental injury during airway management, preoperative dental assessment may also provide clinically relevant information for identifying patients at increased risk of postoperative pneumonia.

In addition to the increased risk of pneumonia [30], patients with unfavorable dental status experienced fewer hospital-free days and ICU-free days within 90 days. These findings suggest that dental status may also be associated with greater healthcare resource utilization [31]. Because postoperative pneumonia may prolong ICU stay, increase antimicrobial exposure, and delay recovery [32], early identification of poor dental status may help clinicians recognize patients at risk for complicated postoperative courses. Based on these findings, we present a practical workflow for incorporating simplified preoperative dental assessment into routine perioperative risk stratification in patients with major burns (Figure 1).

Several limitations should be considered when interpreting our findings. First, this study was conducted at a single burn center, which may limit the generalizability of the results. Second, preoperative dental status was assessed using a simplified clinical examination performed by experienced attending anesthesiologists who routinely assess the oral cavity during preoperative anesthesia evaluations rather than by dental specialists. Because comprehensive dental examinations are often impractical in critically ill burn patients undergoing urgent surgery, this simplified assessment was intended as a pragmatic perioperative screening tool rather than a validated dental index. Third, although the multivariable analysis adjusted for important baseline differences, including age, ASA physical status, inhalation injury, and deep burn area, these factors remain well-established clinical determinants of postoperative pneumonia and may have continued to contribute to pneumonia risk despite statistical adjustment. In particular, inhalation injury is a major risk factor for pulmonary complications following burn injury and may not be fully accounted for despite multivariable adjustment. Therefore, residual confounding from these and other measured or unmeasured factors cannot be completely excluded in this retrospective study. Such unmeasured factors may include pre-existing oral hygiene practices, periodontal disease severity, nutritional status, and socioeconomic factors. Accordingly, although preoperative dental status remained independently associated with postoperative pneumonia after multivariable adjustment, this association should be interpreted with caution and should not be regarded as evidence of a causal relationship. Finally, the observational design of this study precludes the establishment of a causal relationship between preoperative dental status and postoperative pneumonia. Nevertheless, this study included a relatively large cohort of burn patients from a high-volume tertiary burn center, with detailed perioperative data, which strengthens the validity and clinical relevance of our findings. Despite these limitations, our findings suggest that preoperative dental status may represent a simple and clinically accessible marker associated with postoperative pneumonia in burn patients. Future prospective studies incorporating standardized dental assessments, oral microbiome analyses, and interventional oral care protocols are warranted to determine whether improving perioperative oral health can reduce postoperative pulmonary complications in patients with major burns.

## 5. Conclusions

Preoperative dental status was associated with increased 30-day postoperative pneumonia and fewer 90-day hospital-free and ICU-free days in burn patients undergoing surgery. These findings suggest that routine preoperative evaluation of dental status may help identify patients at increased risk of postoperative pulmonary complications. However, prospective studies are needed to validate this simplified assessment approach and determine its role in perioperative risk stratification before it can be incorporated into routine clinical practice.

## Figures and Tables

**Table 1:** Comparison of patient characteristics by pneumonia status.

Variables	Total Patients (*n* = 894)	Non-Pneumonia (*n* = 600)	Pneumonia (*n* = 294)	*p*-Value
Age (years)	54 (44–65)	52 (42–63)	58 (46–69)	<0.001
Sex (male)	724 (81.0)	498 (83.0)	226 (76.9)	0.030
Body mass index (kg/m^2^)	23.5 (21.5–25.7)	23.8 (21.9–26.0)	23.0 (20.8–24.7)	<0.001
ASA physical status				<0.001
≤2	289 (32.3)	226 (37.7)	63 (21.4)	
≥3	605 (67.7)	374 (62.3)	231 (78.6)	
Diabetes mellitus	115 (12.9)	65 (10.8)	50 (17.0)	0.011
Hypertension	239 (26.7)	151 (25.2)	88 (29.9)	0.148
End-stage renal disease	32 (3.6)	18 (3.0)	14 (4.8)	0.185
Burn character				
Burn type				<0.001
Flame, scalding, and contact burn	750 (83.9)	481 (80.2)	269 (91.5)	
Electrical burn	144 (16.1)	119 (19.8)	25 (8.5)	
Inhalation injury	267 (29.9)	144 (24.0)	123 (41.8)	<0.001
Deep burn area	30.0 (20.0–42.0)	28.0 (18.0–40.0)	35.0 (21.3–50.0)	<0.001
Post-burn day (days)	3 (2–5)	3 (2–5)	3 (2–5)	0.229
Unfavorable dental status *	109 (12.2)	57 (9.5)	52 (17.7)	0.001
Preoperative laboratory data				
Hemoglobin (g/dL)	13.2 (10.7–15.7)	13.2 (11.0–15.6)	13.1 (10.3–16.0)	0.625
Albumin (g/dL)	2.6 (2.3–3.0)	2.7 (2.3–3.1)	2.4 (2.1–2.9)	<0.001
Creatinine (mg/dL)	0.7 (0.6–0.9)	0.7 (0.6–0.9)	0.8 (0.6–1.0)	0.008
Operation				
Operation time (min)	80 (55–110)	80 (55–105)	85 (65–115)	0.009
Vasopressor usage	339 (37.9)	201 (33.5)	138 (46.9)	<0.001
RBC transfusion (units)	3.0 (2.0–5.0)	3.0 (2.0–4.0)	3.0 (2.0–5.0)	<0.001
Crystalloid infused (mL/kg)	13.9 (8.6–21.4)	13.1 (8.1–20.7)	16.3 (10.0–22.2)	<0.001
Colloid infused (mL/kg)	6.7 (3.7–10.4)	6.7 (3.7–10.7)	6.7 (3.7–10.1)	0.593
Urine output (mL)	150.0 (80.0–280.0)	150.0 (80.0–280.0)	140.0 (71.3–273.8)	0.538

Continuous variables are described using median and interquartile ranges. Categorical variables are presented as frequencies and percentages. Preoperative laboratory data were measured within 1 day before surgery. * Percentages in Table 1 are column percentages based on postoperative pneumonia status. In contrast, the values of 47.7% and 31.2% shown in Figure 3 represent the incidence of postoperative pneumonia within the unfavorable and favorable dental status groups, respectively. ASA, American Society of Anesthesiologists; RBC, red blood cell.

**Table 2:** Univariate and multivariate logistic regression analyses for risk factors associated with 30-day pneumonia after burn surgery.

Variables	Univariate Analysis		Multivariate Analysis	
	OR (95% CI)	*p*-Value	OR (95% CI)	*p*-Value
Age	1.017 (1.009–1.026)	<0.001	1.021 (1.011–1.032)	<0.001
Sex (male)	0.681 (0.483–0.963)	0.029	0.514 (0.349–0.757)	0.003
ASA physical status		<0.001		0.016
≤2	1.0		1.0	
≥3	2.216 (1.610–3.080)		1.584 (1.120–2.258)	
Diabetes mellitus	1.687 (1.129–2.509)	0.010		
Hypertension	1.244 (0.912–1.696)	0.168		
End-stage renal disease	1.815 (0.893–3.687)	0.099		
Burn character				
Burn type		<0.001		
Flame, scalding and contact burn	1.0			
Electrical burn	0.376 (0.233–0.583)			
Inhalation injury	2.278 (1.691–3.070)	0.001	2.196 (1.591–3.034)	<0.001
Deep burn area	1.022 (1.014–1.031)	<0.001	1.027 (1.017–1.036)	<0.001
Unfavorable dental status	2.047 (1.363–3.070)	0.001	1.681 (1.084–2.599)	0.018
Preoperative laboratory data				
Albumin	0.579 (0.466–0.715)	<0.001	0.770 (0.603–0.994)	0.047
Creatinine	1.600 (1.256–2.095)	<0.001	1.682 (1.289–2.258)	<0.001
Operation				
Vasopressor usage	1.756 (1.363–2.336)	<0.001		
RBC transfusion	2.606 (1.678–4.198)	<0.001		
Crystalloid infused	1.019 (1.008–1.031)	0.001		

Multivariate logistic regression analysis was performed using the forward conditional method. OR, odds ratio; CI, confidence interval; ASA, American Society of Anesthesiologists; RBC, red blood cell.

## Data Availability

The data presented in this study are available on request from the corresponding author. The data are not publicly available due to ethical restrictions.

## Abbreviations

The following abbreviations are used in this manuscript:

ICU	Intensive care unit
RBC	Red blood cell
ASA	American Society of Anesthesiologists
DMFT	Decayed, Missing, and Filled Teeth
CPI	Community Periodontal Index
OHAT	Oral Health Assessment Tool
